# Explicit and Implicit Responses to Tasting Drinks Associated with Different Tasting Experiences

**DOI:** 10.3390/s19204397

**Published:** 2019-10-11

**Authors:** Daisuke Kaneko, Maarten Hogervorst, Alexander Toet, Jan B. F. van Erp, Victor Kallen, Anne-Marie Brouwer

**Affiliations:** 1 Kikkoman Europe R&D Laboratory B.V., 6709 PA Wageningen, The Netherlands; 2 Microbiology and Systems Biology, TNO, 3704 HE Zeist, The Netherlands; victor.kallen@tno.nl; 3 Perceptual and Cognitive Systems, TNO, 3769 DE Soesterberg, The Netherlands; 4 Research Group Human Media Interaction, University of Twente, 7522 NB Enschede, The Netherlands

**Keywords:** explicit measure, implicit measure, behavioral measure, (neuro)physiological measure, food-evoked emotion, discriminative power

## Abstract

Probing food experience or liking through verbal ratings has its shortcomings. We compare explicit ratings to a range of (neuro)physiological and behavioral measures with respect to their performance in distinguishing drinks associated with different emotional experience. Seventy participants tasted and rated the valence and arousal of eight regular drinks and a “ground truth” high-arousal, low-valence vinegar solution. The discriminative power for distinguishing between the vinegar solution and the regular drinks was highest for sip size, followed by valence ratings, arousal ratings, heart rate, skin conductance level, facial expression of “disgust,” pupil diameter, and Electroencephalogram (EEG) frontal alpha asymmetry. Within the regular drinks, a positive correlation was found between rated arousal and heart rate, and a negative correlation between rated arousal and Heart Rate Variability (HRV). Most physiological measures showed consistent temporal patterns over time following the announcement of the drink and taking a sip. This was consistent over all nine drinks, but the peaks were substantially higher for the vinegar solution than for the regular drinks, likely caused by emotion. Our results indicate that implicit variables have the potential to differentiate between drinks associated with different emotional experiences. In addition, this study gives us insight into the physiological temporal response patterns associated with taking a sip.

## 1. Introduction 

Information about food-evoked emotions in addition to simple liking ratings have been argued to improve predictions regarding consumers’ food choices [1,2,3,4,5]. Researchers have developed and used emotion-association questionnaires, in which individuals indicate to what extent they experience certain emotions after tasting foods and/or beverages [6,7,8]. Such explicit self-reporting measures are relatively easy and cost-effective to apply. However, they have inherent drawbacks: they are discontinuous, prone to demand characteristics, may suffer from response biases, and may not cover subconscious processes [9,10]. Furthermore, the “emotional” lexicon varies across cultures and languages, and consumers are not used to verbalizing their emotions, particularly when it comes to foods [2]. Finally, when consumers are asked to explicitly rate their emotions, this can interfere with the food experience itself [11]. Several authors propose to measure unconscious (implicit) responses in addition to self-reports in order to better understand consumers’ food-evoked emotions and predict their future food choice behavior [8,12,13]. These measures can be of a (neuro)physiological nature (e.g., brain signals) or behavioral (e.g., facial expression). The current study aims to provide an overview of the sensitivity of a range of simultaneously measured implicit and explicit measures in response to tasting drinks that are associated with different affective experiences. 

Several non-verbal, implicit measurements have been studied before in the context of probing affective food experience in response to tasting. With respect to facial movements, Steiner et al. investigated affective reactions to pleasant and unpleasant food tastes in human infants and primates [14]. In their experiment, they clearly distinguished between hedonic and aversive expressions without the use of questionnaires. More recently, Danner et al. conducted facial expression analysis elicited by six different fruit and vegetable juices and obtained a negative association of the facial expression “neutral” with disliking and a positive association of “angry” and “disgusted” expressions with disliking, indicating that facial expression analysis may complement self-report questionnaires [15]. Similar results were found in [16] which reported more neutral facial expressions were elicited by liked breakfast drinks compared to less liked ones. 

A few studies examined physiological responses to tasting in the context of affective food experiences. In these studies, participants tasted basic taste solutions, juices, and foods while recording the autonomic nervous system through skin conductance or electrodermal activity (EDA) and heart rate (HR) [15,17,18]. The drinks and foods were also explicitly rated with respect to their liking or pleasantness. They all reported that high heart rate and high EDA was associated with unpleasant ratings. 

In emotions research, emotions are often described not only in terms of pleasantness or valence but also in terms of arousal, or the intensity of the emotion (or (neural) activation) [19,20,21,22,23,24]. While valence and arousal are in principle independent, the drink and food stimuli, as used in References [15,17,18], may have confounded pleasantness (valence) and arousal, where unpleasant food or drinks were high in arousal and vice versa. This would fit with the notion that especially EDA is a reliable indicator of arousal (the sweat glands being innervated by the sympathetic part of the autonomic nervous system) rather than valence [25]. In [16], autonomic nervous system responses elicited by tasting different breakfast drinks were recorded, and participants were not only questioned about liking, but also about intensity. As could be expected, positive correlation coefficients between intensity and EDA were found. There was a negative correlation between intensity and heart rate. In contrast to a high EDA and high heart rate for unpleasant ratings as reported in the studies mentioned above [15,17,18], positive correlation coefficients between liking and EDA, as well as between liking and heart rate, were found in [16]. However, from this paper, it is not clear whether all these correlations were significant. The differences in ratings between drinks were very small. 

As indicated by the literature reviewed above, relating implicit physiological and behavioral measurements to emotional food experience is not straightforward. In general, relations between neurophysiology and emotion depend on both stimuli and context [26,27,28]. It is still an open question as to what extent implicit (neuro)physiological measures, facial expressions, and behavioral measures can be used to monitor emotional food experience relative to, or in addition to, explicit self-report measures, and how they compare to one another. Existing studies used only a few implicit measures, and most asked only for liking or preference scores, or types of emotion, thus omitting the arousal dimension. 

The current study aims to fill these gaps in the literature by simultaneously examining a wide range of implicit measures and by including arousal ratings. Furthermore, we examine the case in which individuals know what they are about to taste. This is usually the case in daily life, but contrasts with most studies that focus on the effect of taste only and therefore do not provide any other information about the drink or food [15,17,18,29]. Finally, while most studies used stimuli either associated with very strong or very subtle differences in emotional experience, we take both approaches in this study. This is done because both approaches have their drawbacks as well as their merits. Since explicit ratings can be biased, it is difficult to assess the “real” emotional experience. This is referred to as the ground truth problem [26]. From this point of view, it is a good choice to use a stimulus that is a priori known to be associated with a strong emotion. This will result in choosing a quite extreme stimulus, such as a quinine solution [14], or in our case, a vinegar solution. On the other hand, we do not know whether findings from studies with an extreme stimulus generalize to more subtle differences. In the current study, we therefore explore whether examining responses to regular drinks with subtle differences in emotional experience on the one hand, and comparisons between regular drinks and a strongly emotional “ground truth” drink on the other hand, lead to similar results with respect to the sensitivity of the investigated variables to reflect emotional food experience. Below, we shortly outline our study and define specific hypotheses.

In the present study, participants were informed what drink they were about to taste before they took a sip of the drink and rated the drink’s valence and arousal. Besides these explicit measures of valence and arousal, we recorded a range of implicit measures while the participants were performing this task: sip size, facial expression of disgust, neurophysiological measures (Electroencephalogram (EEG)), and measures of the autonomic nervous system (pupil size, EDA, and HR). As mentioned above, we take two approaches to probe the sensitivity of these measures of emotional food experience. The first approach is to include a special drink that is expected to be more strongly associated with a certain emotion than the other regular drinks. We chose a drink that evokes a high-arousal, low-valence affective response (a vinegar solution). Comparing the responses to the regular drinks to the response to this generally disliked (“ground truth”) drink indicates the discriminative power of a specific response measure, i.e., how well the different response measures distinguish a strongly disliked drink from regular drinks (with associated emotions that are close to each other). Second, we tested the sensitivity of implicit measures to distinguish responses to the regular drinks that only differed slightly in terms of associated emotions. We use self-reported valence and arousal as generally accepted measures of the affective experience of regular drinks, and correlate each of the remaining measures with these traditional measures. 

We expect the high-arousal, low-valence experience of the vinegar solution to be reflected in the explicit ratings and a smaller sip size. For the rest of our measures, we hypothesize the following associations. Because arousal is consistently and positively related to both pupil size and EDA [25,30], we expect a larger pupil size and increased EDA when tasting the vinegar solution compared with the regular drinks. While no straightforward relation between heart rate and arousal or valence exists across contexts [26], the previous studies suggest a higher heart rate for high arousal in the context of tasting [15,17,18]. We also expect heart rate variability (HRV) to be negatively associated with stress or arousal, as reported in other contexts [31,32]. For valence, we expect the facial expression of disgust to be informative [14,15,16]. Furthermore, we examine frontal brain activation as an indicator of valence. Relatively strong frontal left brain activation has been associated with positive valence and relatively strong right activation with negative valence [33,34]. Research reviewed by Harmon-Jones et al. [35] indicates that rather than valence, frontal brain asymmetry more parsimoniously maps onto approach and avoidance, where relatively strong right frontal brain activation, as indicated by frontal EEG alpha asymmetry, is associated with avoidance and the reverse with approach motivation. In general, negative valence can be associated with both approach (e.g., being angry and wanting to fight) and avoidance motivation (e.g., being scared and wanting to flee). In the food domain, however, high valence can be expected to be associated with approach motivation and low valence with avoidance motivation. We therefore view alpha asymmetry as an indicator of valence. As far as we know, this measure has not been studied in tasting, but there is evidence for its relation with the approach/avoidance motivation or valence coming from studies using food pictures [36,37] and cooking and tasting a chicken versus mealworm dish [33]. 

In sum, the research questions in this study are (i) how well do different self-reported, physiological, and behavioral variables discriminate regular drinks from a drink that is known to be strongly disliked, (ii) how sensitive are these measures to the subtle differences between the regular drinks, and (iii) how are different implicit measures associated with self-reported valence and arousal for the regular drinks? In addition to these research questions, we examine the general temporal pattern of physiological variables after the announcement of the drink and taking a sip.

## 2. Materials and Methods 

### 2.1. Participants 

A total of 70 healthy participants (19 men, 51 women) took part in this study. All of them were of Dutch nationality and were between 19 and 63 years old, with an average of 48.5 years and a standard deviation of 10.5 years. Participants were recruited through the participant pool of the research institute where the study took place (TNO Netherlands Organisation for Applied Scientific Research (TNO)) and received a monetary reward to compensate for time and travel costs. All participants signed an informed consent in accordance with the Helsinki Declaration of 1975, as revised in 2014 [38], before participating in this study. The study was approved by the TNO Institutional Review Board. Three participants were excluded due to technical problems related to the registration of event markers and physiological data recording. This left us with 67 participants for further analysis. For the analysis of facial expression, we only investigated participants without glasses (42).

### 2.2. Materials

#### 2.2.1. Recording Equipment

EDA (for skin conductance level—SCL), ECG (for inter-beat interval—IBI), and EEG (for frontal alpha asymmetry—FAA) were recorded using an Active Two MkII system (Biosemi B.V., Amsterdam, The Netherlands), with a sampling frequency of 512 Hz. SCL was measured by placing gelled electrodes on the fingertips of the index finger and the middle finger of the non-dominant hand. ECG electrodes were placed on the right clavicle and on the lowest floating left rib. For EEG, 32 active silver-chloride electrodes were placed according to the 10–20 system.

Pupil diameter (PD) was recorded at 60 frames per second using SmartEyePro V6.1.6 (Smart Eye AB, Göteburg, Sweden). This system consists of two cameras (Basler acA640-120gm, HR 8.0 mm lens) placed at the left and right side of a screen that presented the name of the drink and the rating scales. The screen had a size of 37.0 by 30.0 cm and the viewing distance was approximately 80.0 cm.

Participants’ faces were recorded using a Color CCTV Camera, WV-CP150E (Panasonic Corp., Osaka, Japan) during the entire experiment. The video camera was positioned at the left side of the screen. 

#### 2.2.2. Self-Report Rating Scales and Sip Size

The self-assessment manikin (SAM) [39] with nine-point scales were used for valence and arousal self-report ratings. The nine-point scale was positioned in the appropriate location at the bottom of each SAM, where the most leftward (most unpleasant and calm) and the most rightward (most pleasant and aroused) parts of the scale corresponded to values of 1 and 9, respectively. With respect to valence, participants were asked how pleasant their experience with the drink was, with the manikin on the right indicating a very pleasant experience and the manikin on the left a very unpleasant experience. With respect to arousal, participants were asked how intensely they experienced the drink, with the manikin on the right indicating a very intense experience and the manikin on the left a very calm experience. Also, they were instructed that they should try to answer quickly, without thinking too long.

For the behavioral measure of sip size, the exact weight of each drink including the cup was measured before the participant took a sip. After finishing the experiment, the cups containing the rest of the drinks were weighted again to determine the sip size.

#### 2.2.3. Samples 

The drinks used in this study were apple juice (Appelsientje), orange juice (Appelsientje), yogurt drink (Vifit), milk (Campina), buttermilk (Campina), rooibos tea (Pickwick), black tea (Pickwick), cola (Coca-cola), and diluted vinegar (Private Brand of Plus: 50% vinegar, 50% water) solution. The regular drinks were chosen to represent a variation in basic flavors and temperature. They differed from one another in taste, but they were expected to be close to one another in affective experience, at least relative to diluted vinegar. Teas and the vinegar solution were always prepared in the same way each morning. Teas were kept at about 60 °C, and the vinegar solution was kept at room temperature. The other drinks were kept in a refrigerator before being served to the participants. Each sample was served in a white plain cup, in portions of 50 g. Participants tasted the drinks in randomized order except for a 50 g cup of water, which was always presented after the vinegar solution to decrease the possible lingering of emotional and physiological effects. Responses to water are not included in the analyses. 

### 2.3. Design and Procedure 

**Figure 1:**
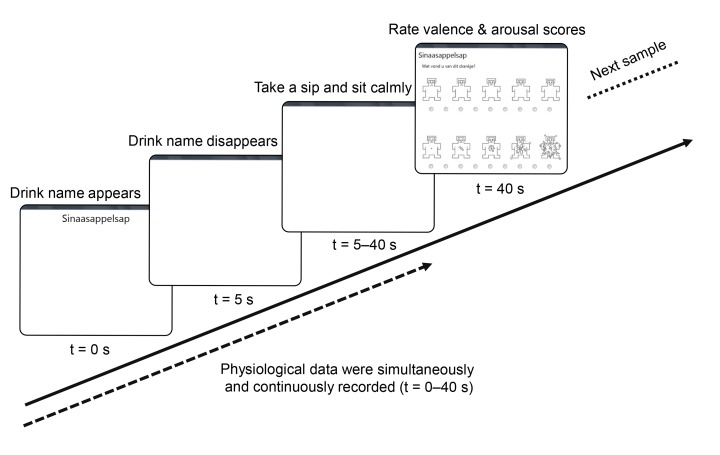
Schematic overview of an experimental trial. Participants clicked the small circles below the SAM scales in order to give their response, after which, a new trial started.

After participants arrived at the laboratory, the experimental procedure was explained, and they were asked to sign the informed consent form. The electrodes for EDA, ECG, and EEG were attached, and participants were asked to sit comfortably in front of the screen. The EDA electrodes were worn on the non-dominant hand, and participants were asked to pick up the sample cups with their dominant hand. Participants filled out a general questionnaire on demographic details and current emotional state. Before the experiment started, the SmartEyePro system was calibrated. Then, the experimenter showed and explained how to take a sip, immediately putting the cup down after the sip, and participants performed a practice trial with water. After this there was time for additional practice or instructions when needed, and participants had the chance to ask questions. The timeline of an experimental trial is indicated in Figure 1. First, the name of the drink was presented on the screen. This was the sign for the experimenter to place the appropriate drink in front of the participant. After 5 seconds, the name of the drink disappeared, which was the sign for participants to take one sip. 

After taking the sip, participants sat still and looked at a blank white screen. Forty seconds after the name of the drink had appeared on the screen, the self-report valence and arousal rating scales appeared, with the valence scale on top. After rating valence and arousal, the name of the next drink appeared on the screen. This procedure was repeated until all drinks had been served.

### 2.4. Data Processing and Analysis

The analysis performed on the physiological data for EDA, ECG, and EEG were similar to analyses in the previous studies [33,40,41].

#### 2.4.1. Preprocessing for Facial Expression, EDA, ECG, and EEG

For the analysis of the facial expression of disgust (FR_disgust_), the video data was analyzed using FaceReader software version 7.0 (Noldus Information Technology B.V., Wageningen, The Netherlands) at a sampling rate of 12.5 frames per second. FaceReader extracts the basic emotional expressions, including disgust, using an artificial neural network that was trained on over 10,000 pictures and exploiting a number of facial features, including facial action units, gaze direction, and head orientation. Calibration procedures were conducted for each participant to correct for person-specific biases toward a certain facial expression according to the FaceReader manual. FR_disgust_ is expressed as a value from 0 to 1 in each frame, indicating the intensity of the emotion. “0” means that the emotion is not visible in the facial expression, “1” means that the emotion is fully present.

The EDA signal was bandpass filtered between 0.03 and 100 Hz. Inter-beat interval (IBI), defined as the temporal distance between R-spikes [42], was extracted from the ECG signal using custom made MATLAB 2019a (www.mathworks.com) algorithms. 

Raw EEG data were pre-processed and analyzed using MATLAB and the FieldTrip open source MATLAB toolbox [43]. The EEG pre-processing entailed standard procedures of referencing the signals to the average EEG signal and filtering them using a 0.5 Hz high pass and a 43 Hz low pass filter to remove slow drifts and high-frequency noise. Logistic infomax independent component analysis (ICA, [44]) was performed to classify artifactual independent components, i.e., components not reflecting sources of neural activity, but were rather ocular or muscle-related artifacts. These components were removed from the data. This was done using EEGLAB v14.1.2 for MATLAB [45]. Measurement intervals, starting at the onset of the announcement of the drink and ending 40 seconds later (at the time that the rating scales appeared), were divided into 5 s intervals. For each of these intervals, the spectral power was calculated over bands ranging from 8 to 13 Hz (alpha) in steps of 0.2 Hz following a fast Fourier transform (FFT) approach using a single Hanning taper. Subsequently, values were integrated. FAA at F7 and F8 was determined for each 5-s segment by taking the relative difference between alpha as recorded at the right and the left side of the cortex: ((R − L)/(R + L)) × 100 [46]. Positive values indicate lower alpha power in the left than in the right hemisphere (i.e., relatively greater left hemisphere cortical activity).

#### 2.4.2. Extraction of Variables

For each of the variables (valence, arousal, sip size, FR_disgust_, SCL, IBI, HRV, PD, and FAA), we required one value for each participant and each drink. For rated valence, rated arousal, and sip size, one value was already present. For the continuously measured variables, these values were extracted as follows: For FR_disgust_ and IBI, we averaged values across the forty seconds starting at onset of the announcement of the drink and ending 40 seconds later. Since PD and FAA were rather noisy, we used the median across the 40 seconds rather than the mean. HRV was calculated as the root of the mean of squared successive differences of the IBIs (RMSSD) across the 40 seconds (i.e., the average absolute difference between successive IBIs [47,48]). SCL showed strong drifts across the duration of the experiment. Therefore, before taking the average across the 40 second intervals, SCL curves were baselined using the average of the first 5 seconds of data. Subsequently, sip size, SCL, IBI, and PD were log transformed, leading to more normal distributions.

To remove irrelevant overall differences between participants, we centered the data by subtracting the mean value for each variable and each participant. 

For each variable, before and after this subtraction, values that were more than five standard deviations away from the mean were discarded as outliers. For FAA, this was also done for the alpha values that were used to compute the FAA. This procedure led to 9.9% lost data for HRV, 5.3% lost data for sip size, 10.4% lost data for FAA, and less than 2.0% of data loss for all other variables.

To examine how the different physiological variables evolved over time, we also determined a value for each participant and drink in the same way as described above, but for each subsequent 5-s interval rather than the whole 40 s. To visualize potential differences in patterns between drinks clearly, we baselined the curves for each drink using the first 5 s of the data. This was not done for HRV, since 5 s was too short an interval to obtain HRV in a meaningful way. 

#### 2.4.3. Statistical Analyses

To examine how well different variables discriminated regular drinks from the vinegar solution, we calculated one z-score (z) for each variable using the equation below:

(1) $$z=\frac{\left(\mathrm{mean}\;\mathrm{value}\;\mathrm{regular}\;\mathrm{drinks}\right)-\left(\mathrm{mean}\;\mathrm{value}\;\mathrm{vinegar}\;\mathrm{solution}\right)}{\sigma_{diff}}$$

To calculate sigma (σ*_diff_*), we started with the distribution of the values in response to the vinegar solution, and the distribution of the values in response to all other eight drinks. The standard deviation of the distribution of the differences between these two was estimated by taking the width of the 95% confidence interval of the difference and dividing this by 4. The z-score (or discriminative power) was significant (at *p* < 0.05) when larger than 1.96.

Next, we performed individual one-way ANOVAs for each variable with the regular drink as the independent variable (eight regular drinks). These tests indicated how sensitive the measures were to the subtle differences between the regular drinks. While a large F-value and a low *p*-value were indicative of a sensitive measure, it would make a difference whether this hinged upon only one or several significant comparisons. Therefore, for the measures that showed a significant effect for a “regular drink”, we also report how many and which of the total possible number of 28 pairwise post-hoc comparisons between regular drinks reached significance (Tukey’s HSD).

To investigate the association between different implicit measures and self-reported valence and arousal for the regular drinks, we calculated (for each implicit measure separately) the correlation between the implicit measures and both valence and arousal. We used the scores averaged per drink as input, i.e., one implicit value and one rating score per drink, resulting in eight data pairs. This analysis explored whether there was a systematic ordering of the drinks along both dependent variables.

## 3. Results 

### 3.1. Sensitivity of Measures to Distinguish between Regular Drinks and Diluted Vinegar

**Figure 2:**
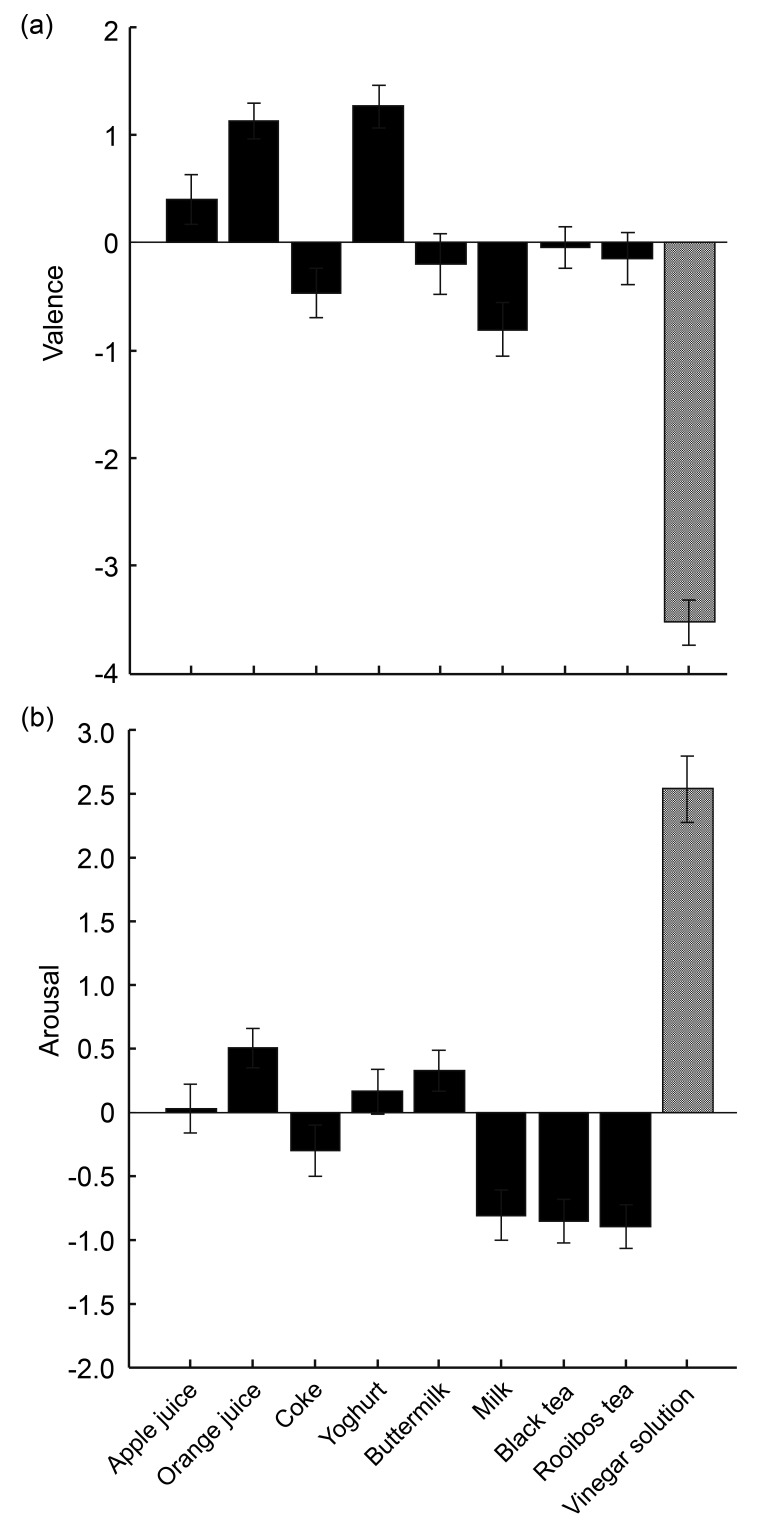
The results of explicit ratings: valence (**a**) and arousal (**b**) averaged across participants. Solid bars represent the eight regular drinks, and the dotted bar represents the vinegar solution.

Reported valence and arousal of each drink, averaged across participants, are shown in Figure 2. The valence and arousal ratings of the vinegar solution were on average the lowest and the highest of all drinks tested, respectively. These results are in accordance with our assumption that the vinegar solution could serve as the ground truth unpleasant and arousing drink. 

**Figure 3:**
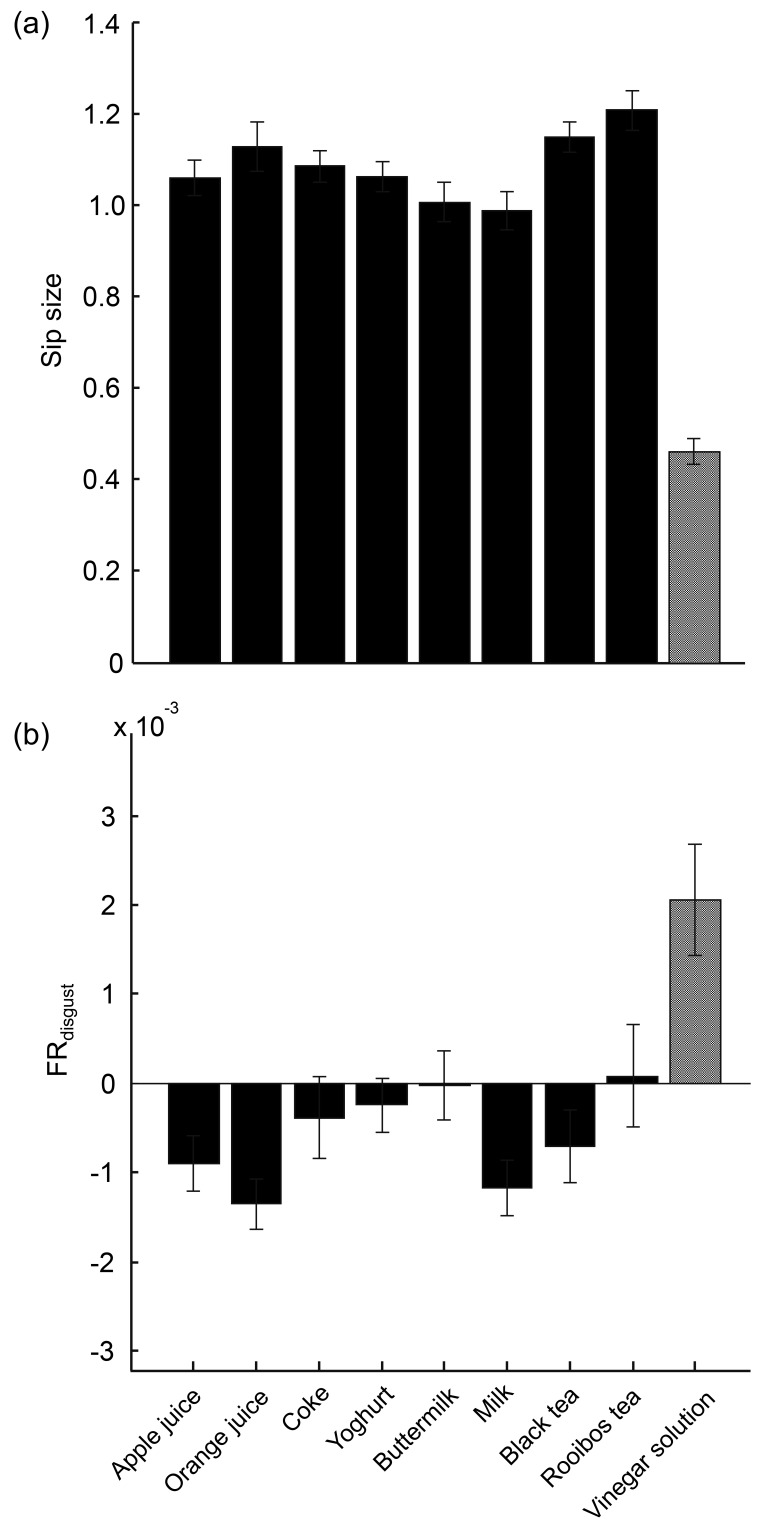
The results of behavioral measures: sip size (**a**) and FR_disgust_ (**b**) averaged across participants. Solid bars represent the eight regular drinks, and the dotted bar represents the vinegar solution.

The implicit behavioral measures, sip size, and FR_disgust_ are shown in Figure 3, separately for each drink and averaged across participants. The sip size of the vinegar solution was the smallest, and the FR_disgust_ was the highest.

**Figure 4:**
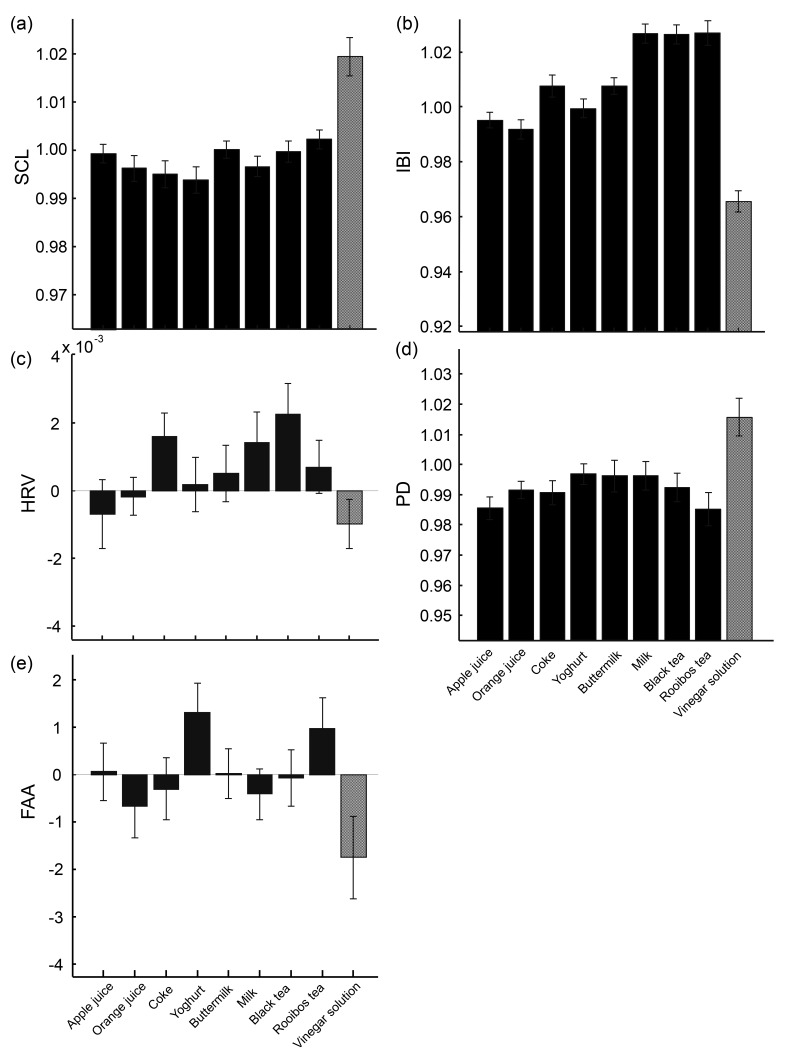
The results of physiological measures: SCL (**a**), IBI (**b**), HRV (**c**), PD (**d**), and FAA (**e**) averaged across participants. Solid bars represent the eight regular drinks, and the dotted bar represents the vinegar solution.

Figure 4 shows SCL, IBI, HRV, PD, and FAA for each drink averaged over participants. All measures show the most extreme value for the vinegar solution in the expected direction: it was the highest of all drinks for SCL and PD, and the lowest for IBI, HRV, and FAA (low FAA indicating a higher alpha power in the left rather than in the right hemisphere, i.e., relatively greater right hemisphere cortical activity, consistent with negative valence or avoidance).

The discriminative power (z-score) to distinguish vinegar from regular drinks is presented in Table 1. Sip size had the highest discriminative power of all measures, followed by the explicit ratings of valence and arousal, and IBI, all with z-scores higher than 10. FR_disgust_, SCL, and PD had z-scores between 4 and 8, indicating highly significant discriminative power. For HRV, the z-score was below 1.96, indicating that the discriminative power of this measure to distinguish between the vinegar solution and the regular drinks was too low to reach significance. 

### 3.2. Sensitivity of Measures to Distinguish between Regular Drinks

For each separate measure, a one-way repeated measures ANOVA with regular drink as independent variable was conducted to evaluate its sensitivity to distinguish between regular drinks. In these analyses, data associated with tasting the vinegar solution were left out. The results are summarized in Table 2. Valence and arousal ratings, sip size, and IBI showed significant responses regarding regular drinks. FR_disgust_, SCL, HRV, PD, and FAA did not differ significantly between drinks. The sensitivity of the measures as indicated by the F- and *p*-values were paralleled by the number of significant comparisons, as indicated by the post hoc tests. Among all 28 possible pairwise combinations of the 8 regular drinks, 11, 13, 2, and 17 combinations were significantly different to each other in terms of valence, arousal, sip size, and IBI, respectively. Table 3 presents which pairs of drinks differed for which measure.

### 3.3. Association between Implicit Measures and Self-Reported Valence and Arousal for Regular Drinks

**Figure 5:**
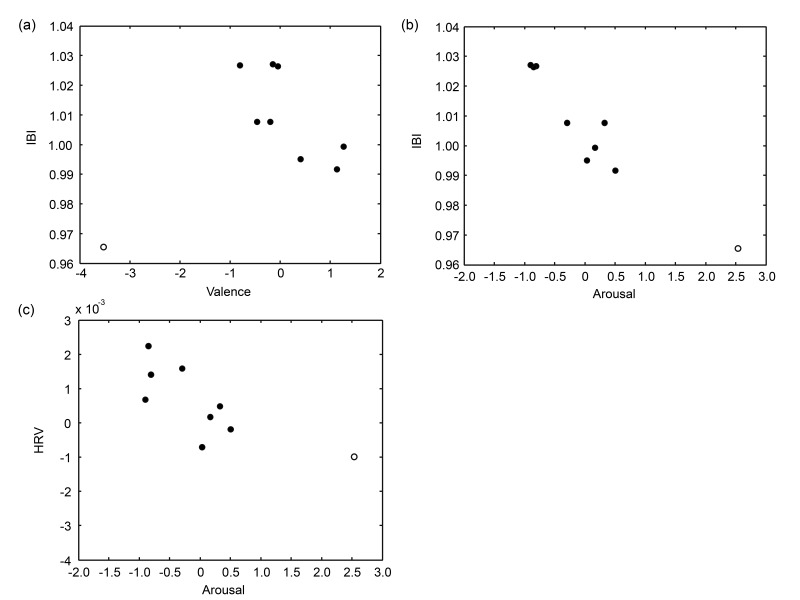
The data plots displaying the significant correlations between (**a**) valence and IBI, (**b**) arousal and IBI, and (**c**) arousal and HRV. Solid circles represent the eight regular drinks, and the open circle represents the vinegar solution. Note that correlation analysis were performed on the eight regular drinks only.

Table 4 summarizes the results of the correlation analyses. Cells where we hypothesized a correlation based on the literature (see Introduction) are highlighted in grey. Valence was significantly correlated with IBI. Arousal was significantly correlated with IBI and HRV. Figure 5 presents the data underlying the three significant correlations.

### 3.4. General Temporal Pattern of Behavioral and Physiological Variables Associated with Taking a Sip

**Figure 6:**
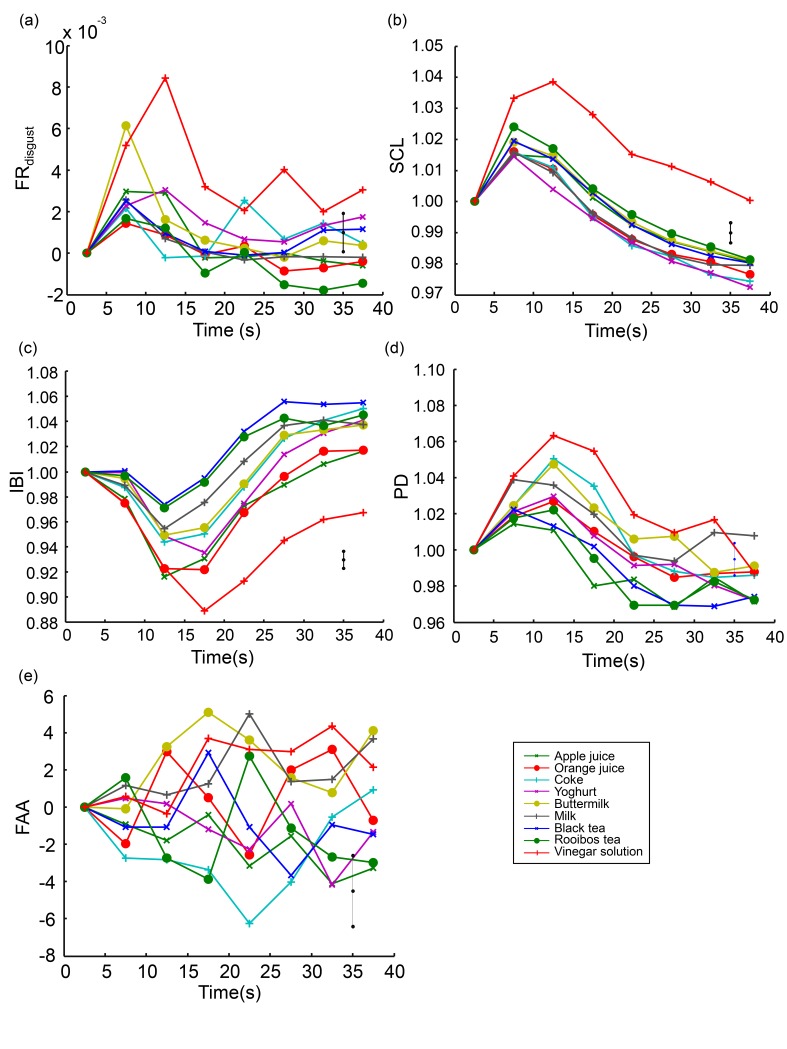
The continuous behavioral measure FR_disgust_ (**a**) and the physiological measures SCL (**b**), IBI (**c**), PD (**d**), and FAA (**e**) plotted over time from the time that the name of the drink appeared (t = 0 s) to just before the rating scales appeared (t = 40 s), averaged across participants and separately for each drink.

The continuous measures (FR_disgust_, SCL, IBI, PD, and FAA) are plotted over time in Figure 6. The onset of the announcement of the drinks occurred at t = 0 s, and the rating scales appeared at t = 40 s. Consistent patterns arose for almost all variables. Every time a participant started taking a sip (t = 5 s), FR_disgust_, SCL, and PD increased, and IBI decreased for all drinks. These patterns could be partly due to effects of movements and ingestion processes associated with picking up a cup, taking a sip, and putting it down. However, consistent with the results presented in the preceding sections, these increases and decreases were clearly stronger for the vinegar solution compared to the regular drinks. For FAA, we did not see a consistent pattern over time. The difference between the vinegar solution and the regular drinks in FAA arose immediately after presentation of the name of the drink. Thus, after aligning the curves at t = 0, the vinegar solution does not stand out as it does for the other continuous measures presented in Figure 6.

## 4. Discussion

The present study evaluated nine different measures of emotional food experience: explicit measures (valence and arousal ratings), implicit behavioral measures (sip size and facial expression of disgust), implicit physiological measures (SCL, IBI, HRV, and PD), and an implicit neurophysiological measure (FAA). We recorded these measures while participants took sips of eight different regular drinks, and one non-regular drink (diluted vinegar). The vinegar solution was expected to differ strongly from the regular drinks in the associated emotional experience by producing the lowest valence and the highest arousal. Our data indeed showed that participants rated the vinegar solution lowest in valence and highest in arousal, took the smallest sip, and showed the most outspoken signs of disgust in their facial expression. We also found, as expected, that the vinegar solution led to higher SCL and PD, lower IBI and HRV, and FAA in the direction of avoidance (negative valence) when compared to the regular drinks. 

The first research question of this study concerned the extent to which explicit and implicit measures could discriminate the vinegar solution (as a ground truth high-arousal, low-valence drink) from the regular drinks. We used z-scores as an index of discriminative power. Sip size had the highest discriminative power, even higher than the explicit valence and arousal ratings. IBI and SCL also showed high discriminative power. Although the scores for FR_disgust_ and PD are somewhat lower, they are still highly significant. FAA seemed to be a less strongly discriminative measure than FR_disgust_ and PD, but it still reliably distinguished between the regular drinks and the vinegar solution. Only HRV did not significantly discriminate between the vinegar solution and the regular drinks. Thus, in addition to explicit ratings, a range of implicit measures could be useful parameters to measure individual’s emotions evoked by a food experience, at least for cases in which food experiences differ strongly. 

For regular drinks, the effect of a drink in the ANOVAs on explicit ratings of valence and arousal suggested that participants also agreed on small differences in affective experience. This enabled us to answer our second research question about the sensitivity of the different measures to reflect subtle differences in affective experience. ANOVAs on sip size and IBI showed that, like explicit ratings, these are sensitive measures as well. FR_disgust_ and SCL did not reach significance, and HRV, PD, and FAA were also not sensitive enough to detect the minor differences between the regular drinks. These results are in line with the results on discriminative power to separate the vinegar solution from the other drinks as discussed above.

The third research question concerned the association between implicit measures and self-reported explicit ratings of the regular drinks. The correlation analyses on the average scores for each drink revealed significant and high correlations (*ρ* > 0.70, explained variance > 50%) between IBI and both valence and arousal, and between HRV and arousal. The correlations between IBI and arousal, and between HRV and arousal, were the correlations that we expected to find based on the literature, and they were in the expected direction. Remarkably, HRV showed significant correlations with explicit ratings while it did not show the effects of each drink tested through the ANOVAs, in which explicit ratings were not taken into account. The fact that HRV did not distinguish well between the vinegar solution and the regular drinks, while this measure did correlate with the explicit rating of arousal for the regular drinks, can be understood when observing the position of vinegar solution in the scatter plot (Figure 5c): the relation between HRV and the explicit measure of arousal did not extend from the regular drinks to vinegar solution. Thus, in contrast to IBI where the relation between explicit arousal and IBI extended from the regular drinks to the vinegar solution (Figure 5b), HRV did not seem to be a valid marker of the affective experience of drinks that are associated with extreme levels of affective experience. 

Fourth, we examined the response pattern over time of the continuous measures to provide insight into the specific patterns before, during, and after tasting. This is important in the context of extracting dependent physiological and facial expression variables and designing research on tasting that includes physiological measures. For this, we needed to know what interval length was suitable to examine physiological data relative to the time of a sip, and how much time should preferably be allowed between sips. 

We found that being presented with the name of a drink and taking a sip produces characteristic patterns in most continuous variables for all nine drinks. This may be due to movement and ingestion related processes, and affective components that may always occur when taking a sip in the experiment. The distinction between the regular drinks and the ground truth high-arousal, low-valence vinegar solution was not reflected in the pattern itself but in the fact that the pattern was more distinct for the vinegar solution compared to the regular drinks: a stronger increase in FR_disgust_, SCL, and PD, and a stronger decrease in IBI. We found that it took 10 to 15 seconds for these differences to fully develop. Few previous studies examined the pattern of physiological variables over time following a sip. Rousmans et al. show a few example traces of EDA and HR following the intake of a taste solution [17], and de Wijk et al. show patterns averaged across participant per breakfast drink over 8 seconds following the instruction to take a sip [16]. In both studies, HR increased and then decreased again. For EDA, de Wijk et al. show a decrease across 8 seconds, while Rousmans et al. reports an increase. 

The general increase in HR (i.e., the decrease in IBI) after taking a sip that we found here was smaller than that found in [16,17]. De Wijk et al. show increases of about 12 bpm for all drinks [16]. In [17], increases vary between 1.3 (water) and 11 (quinine sulfate). In [16], movements were minimized more than in [17] and the current study. The participants in [16] sat still with a straw in their mouth and on a signal, took a sip and kept sitting still until it was indicated that they could take the straw out of their mouth. In [17], participants took a sip from a cup themselves, similar as our participants. The fact that the strongest increases were found in an experiment where participants sat relatively still suggests that the general increase of HR does not seem to be mainly caused by the movement of the hand (e.g., holding a cup and taking a sip). 

We evaluated nine explicit and implicit potential measures of affective food experience. For all of them, we found at least some evidence of their sensitivity. The nine measures differ in several ways and the preferred (combination of) measure(s) will depend on the research question at hand. Explicit valence and arousal ratings have a good ability to measure both large and subtle differences in emotions, but have several disadvantages, as described in the introduction. Sip size had the largest power to discriminate the ground truth of a low-valence and high-arousal vinegar solution from the regular drinks. Although it is not a continuous measure, it taps into implicit behavior and may thus be less prone to response biases than the explicit ratings of valence and arousal. IBI has the advantage of being both continuous and implicit, and did consistently well in all tests; it appeared to be sensitive to both large and subtle differences in affective experience. The correlation between valence and IBI for the regular drinks indicated a high HR for high valence, adding to the mixed findings on associations between HR and valence in tasting studies, as discussed in the introduction. We argue that rather than a relation between HR and valence, the relation in this context was actually between HR and arousal. The correlation between arousal and IBI is much stronger than between valence and IBI, and valence and arousal were not independent in the stimuli we used. We substantiated this idea by performing additional partial correlation analyses, showing that the correlation between IBI and valence disappeared when controlling for arousal, while the correlation between IBI and arousal remained when controlling for valence. FR_disgust_, SCL, and FAA may not be sensitive enough to easily identify subtle differences but were definitely able to discriminate large differences. Apart from being continuous, they were not correlated to the ratings of valence and arousal for regular drinks, and may be considered as tapping into a fundamentally different dimension than these explicit measures. Finally, HRV turned out to be not suitable to discriminate between vinegar solution and regular drinks. However, when analyzing the regular drinks without considering the vinegar solution, HRV did show a significant correlation with explicit ratings of respectively arousal and valence, and may help to increase the validity and reliability of rank ordering regular drinks with subtle emotional differences along arousal and valence scales.

There are some limitations in this study. For each of the physiological and facial expression data streams, choices were made as to which variable to extract and in what way. We aimed to represent each data stream by one (a priori) promising variable, but it may be that other extraction methods or variables show different (better) results. Also, adding a long resting baseline after answering the questionnaire and before the appearance of the name of the drink may have resulted in less noise and higher sensitivity of the physiological variables. In our experiment, participants were asked to take a sip themselves in an effort to enhance the naturalness of tasting. However, the downside of this is added noise through movement and short, partial occlusion of the face when the cup is at the mouth. We here examined the situation that participants tasted a drink that they expected to taste (as is common in daily life). The food experience and physiological processes that we examined were therefore the result of a mixture of expectation and sensory processes, starting at the moment that the name of the drink appears on the screen. We refer to Verastegui-Tena and colleagues for physiological studies that specifically look at the role of expectation in tasting [12]. A final limitation we want to mention is the fact that rated valence and arousal of the regular drinks correlated positively; including regular drinks that are high in arousal and low in valence, or low in arousal and high in valence, would help to disentangle valence and arousal effects.

## 5. Conclusions 

In this study, we tested regular drinks varying in sweetness, carbonation, temperature, sourness, and thickness that were expected to differ slightly with respect to associated affective experience, as well as one “ground truth” low-valence, high-arousal drink, to evaluate the potential of different explicit and implicit measures to reflect food experience. This resulted in a comprehensive overview of the sensitivity of each of the measures to reflect different affective food experiences strongly, as well as more subtle differences. Furthermore, we showed the association between explicit measures and different implicit measures. Out of the complete set of implicit measures (sip size, facial expression of disgust, skin conductance level, heart rate, heart rate variability, and EEG frontal asymmetry), heart rate showed good sensitivity in all cases. We argue that heart rate should be viewed as a measure of arousal rather than valence. Finally, we provided insight into the development of continuous implicit variables over time after taking a sip of drinks differing in affective experience. Our results may guide the design of future studies and applications utilizing implicit measures for quantifying affective experience, which may ultimately enable the continuous monitoring of food experience without influencing the experience itself.

## Figures and Tables

**Table 1:** Z-scores of each measure between vinegar solution and the regular drinks.

Measures	Discrimination Power: Vinegar vs. Regular Drinks (z)
Valence	14.84
Arousal	13.42
Sip size	19.50
FR_disgust_	5.97
SCL	7.97
IBI	10.99
HRV	1.94
PD	4.33
FAA	2.63

**Table 2:** F and *p*-values of each measure with ANOVAs reflecting the effect of the eight regular drinks.

Measures	Regular Drink Sensitivity (F)	Regular Drink Sensitivity (*p*)
Valence	10.6	<0.001
Arousal	10.1	<0.001
Sip size	3.10	0.003
FR_disgust_	1.83	0.080
SCL	1.51	0.162
IBI	17.0	<0.001
HRV	1.31	0.244
PD	0.87	0.532
FAA	1.16	0.327

**Table 3:** Combinations of regular drinks that were significantly different (*p* < 0.05) according the post-hoc tests in terms of valence (“V”), arousal (“A”), sip size (“S”), and IBI (“I”). Examples of no significant differences for any of the four measures is indicated with “ns”.

	Apple Juice	Orange Juice	Milk	Buttermilk	Yogurt Drink	Coke	Rooibos Tea	Black Tea
**Apple Juice**	-	ns	V, A, I	ns	ns	ns	A, I	A, I
**Orange Juice**		-	V, A, I	V, I	ns	V, A, I	V, A, I	V, A, I
**Milk**			-	A, I	V, A, I	I	S	ns
**Buttermilk**				-	V	ns	A, S, I	A, I
**Yogurt Drink**					-	V	V, A, I	V, A, I
**Coke**						-	I	I
**Rooibos Tea**							-	ns
**Black Tea**								-

**Table 4:** The summarized correlation analysis between explicit ratings (valence and arousal) and behavioral (sip size and FR_disgust_) and physiological measures (SCL, IBI, HRV, PD, and FAA). The cells highlighted in light gray represent the correlations we hypothesized based on the physiological literature. The bold data represent significant correlations (*p* < 0.05).

	Valence		Arousal	
	*ρ*	*p*-Value	*ρ*	*p*-Value
Sip size	0.2290	0.585	−0.3051	0.462
FR_disgust_	−0.2285	0.586	−0.0720	0.866
SCL	−0.3488	0.397	−0.3718	0.365
IBI	**−0.7326**	**0.039**	**−0.9225**	**0.001**
HRV	−0.6413	0.087	**−0.7093**	**0.049**
PD	0.0189	0.965	0.2210	0.599
FAA	0.3570	0.385	0.6180	0.102

## References

[1] Thomson D.M.H., Crocker C., Marketo C.G. (2010). Linking sensory characteristics to emotions: An example using dark chocolate. Food Qual. Prefer..

[2] Gutjar S., de Graaf C., Kooijman V., de Wijk R.A., Nys A., ter Horst G.J., Jager G. (2015). The role of emotions in food choice and liking. Food Res. Int..

[3] Dalenberg J.R., Gutjar S., Ter Horst G.J., de Graaf K., Renken R.J., Jager G. (2014). Evoked emotions predict food choice. PLoS ONE.

[4] Cardello A.V., Meiselman H.L., Schutz H.G., Craig C., Given Z., Lesher L.L., Eicher S. (2012). Measuring emotional responses to foods and food names using questionnaires. Food Qual. Prefer..

[5] Kuenzel J., Zandstra E.H., Lion R., Blanchette I., Thomas A., El-Deredy W. (2010). Conditioning unfamiliar and familiar flavours to specific positive emotions. Food Qual. Prefer..

[6] Kenney E., Adhikari K. (2016). Recent developments in identifying and quantifying emotions during food consumption. J. Sci. Food Agric..

[7] Schifferstein H.N. (2015). Employing consumer research for creating new and engaging food experiences in a changing world. Curr. Opin. Food Sci..

[8] Köster E.P., Mojet J. (2015). From mood to food and from food to mood: A psychological perspective on the measurement of food-related emotions in consumer research. Food Res. Int..

[9] Kaneko D., Toet A., Brouwer A.-M., Kallen V., Van Erp J.B. (2018). Methods for evaluating emotions evoked by food experiences: A literature review. Front. Psychol..

[10] Venkatraman V., Dimoka A., Pavlou P.A., Vo K., Hampton W., Bollinger B., Hershfield H.E., Ishihara M., Winer R.S. (2015). Predicting advertising success beyond traditional measures: New insights from neurophysiological methods and market response modeling. J. Mark. Res..

[11] Winkielman P., Berridge K., Sher S. (2011). Emotion, consciousness, and social behavior. Handbook of Social Neuroscience.

[12] Verastegui-Tena L., Schulte-Holierhoek A., Van Trijp H., Piqueras-Fiszman B. (2017). Beyond expectations: The responses of the autonomic nervous system to visual food cues. Physiol. Behav..

[13] Beyts C., Chaya C., Dehrmann F., James S., Smart K., Hort J. (2017). A comparison of self-reported emotional and implicit responses to aromas in beer. Food Qual. Prefer..

[14] Steiner J.E., Glaser D., Hawilo M.E., Berridge K.C. (2001). Comparative expression of hedonic impact: Affective reactions to taste by human infants and other primates. Neurosci. Biobehav. Rev..

[15] Danner L., Sidorkina L., Joechl M., Duerrschmid K. (2014). Make a face! Implicit and explicit measurement of facial expressions elicited by orange juices using face reading technology. Food Qual. Prefer..

[16] De Wijk R.A., He W., Mensink M.G.J., Verhoeven R.H.G., de Graaf C. (2014). ANS responses and facial expressions differentiate between the taste of commercial breakfast drinks. PLoS ONE.

[17] Rousmans S., Robin O., Dittmar A., Vernet-Maury E. (2000). Autonomic nervous system responses associated with primary tastes. Chem. Senses.

[18] De Wijk R.A., Kooijman V., Verhoeven R.H.G., Holthuysen N.T.E., de Graaf C. (2012). Autonomic nervous system responses on and facial expressions to the sight, smell, and taste of liked and disliked foods. Food Qual. Prefer..

[19] Berridge K.C. (1996). Food reward: Brain substrates of wanting and liking. Neurosci. Biobehav. Rev..

[20] Berridge K.C., Ho C.-Y., Richard J.M., DiFeliceantonio A.G. (2010). The tempted brain eats: Pleasure and desire circuits in obesity and eating disorders. Brain Res..

[21] Lang P.J. (1995). The emotion probe: Studies of motivation and attention. Am. Psychol..

[22] Piqueras-Fiszman B., Kraus A.A., Spence C. (2014). “Yummy” versus “Yucky”! Explicit and implicit approach–avoidance motivations towards appealing and disgusting foods. Appetite.

[23] Russell J.A., Pratt G. (1980). A description of the affective quality attributed to environments. J. Pers. Soc. Psychol..

[24] Vansteelandt K., Claes L., Muehlenkamp J., De Cuyper K., Lemmens J., Probst M., Vanderlinden J., Pieters G. (2013). Variability in affective activation predicts non-suicidal self-injury in eating disorders. Eur. Eating Disorders Rev..

[25] Roth W.T. (1983). 8 A Comparison of P300 and Skin Conductance Response. Advances in Psychology.

[26] Brouwer A., Zander T., van Erp J., Korteling J., Bronkhorst A. (2015). Using neurophysiological signals that reflect cognitive or affective state: Six recommendations to avoid common pitfalls. Front. Neurosci..

[27] Cacioppo J.T., Gardner W.L. (1999). Emotion. Annu. Rev. Psychol..

[28] Kreibig S.D. (2010). Autonomic nervous system activity in emotion: A review. Biol. Psychol..

[29] Gomes L.M., Silva R.G., Melo M., Silva N.N., Vanderlei F.M., Garner D.M., de Abreu L.C., Valenti V.E. (2016). Effects of Effortful Swallow on Cardiac Autonomic Regulation. Dysphagia.

[30] Bradley M.M., Greenwald M.K., Petry M.C., Lang P.J. (1992). Remembering pictures: Pleasure and arousal in memory. J. Exp. Psychol. Learn. Mem. Cogn..

[31] Grossman P., Taylor E.W. (2007). Toward understanding respiratory sinus arrhythmia: Relations to cardiac vagal tone, evolution and biobehavioral functions. Biol. Psychol..

[32] Berntson G.G., Thomas Bigger J., Eckberg D.L., Grossman P., Kaufmann P.G., Malik M., Nagaraja H.N., Porges S.W., Saul J.P., Stone P.H. (1997). Heart rate variability: Origins, methods, and interpretive caveats. Psychophysiology.

[33] Brouwer A., Hogervorst M., Grootjen M., van Erp J., Zandstra E. (2017). Neurophysiological responses during cooking food associated with different emotions. Food Qual. Prefer..

[34] Ohgami Y., Kotani Y., Tsukamoto T., Omura K., Inoue Y., Aihara Y., Nakayama M. (2006). Effects of monetary reward and punishment on stimulus-preceding negativity. Psychophysiology.

[35] Harmon-Jones E., Gable P.A., Peterson C.K. (2010). The role of asymmetric frontal cortical activity in emotion-related phenomena: A review and update. Biol. Psychol..

[36] Harmon-Jones E., Gable P.A. (2009). Neural activity underlying the effect of approach-motivated positive affect on narrowed attention. Psychol. Sci..

[37] Gable P., Harmon-Jones E. (2008). Relative left frontal activation to appetitive stimuli: Considering the role of individual differences. Psychophysiology.

[38] WorldMedicalAssociation (2014). World Medical Association Declaration of Helsinki: Ethical principles for medical research involving human subjects. J. Korean Med. Assoc..

[39] Bradley M.M., Lang P.J. (1994). Measuring emotion: The self-assessment manikin and the semantic differential. J. Behav. Ther. Exp. Psychiatry.

[40] Brouwer A., Hogervorst M., Reuderink B., van der Werf Y., van Erp J. (2015). Physiological signals distinguish between reading emotional and non-emotional sections in a novel. Brain-Comput. Interfaces.

[41] Hogervorst M.A., Brouwer A.-M., Van Erp J.B. (2014). Combining and comparing EEG, peripheral physiology and eye-related measures for the assessment of mental workload. Front. Neurosci..

[42] Appelhans B.M., Luecken L.J. (2006). Heart rate variability as an index of regulated emotional responding. Rev. Gen. Psychol..

[43] Oostenveld R., Fries P., Maris E., Schoffelen J.-M. (2011). FieldTrip: Open source software for advanced analysis of MEG, EEG, and invasive electrophysiological data. Comput. Intell. Neurosci..

[44] Bell A.J., Sejnowski T.J. (1995). An information-maximization approach to blind separation and blind deconvolution. Neural Comput..

[45] Delorme A., Makeig S. (2004). EEGLAB: An open source toolbox for analysis of single-trial EEG dynamics including independent component analysis. J. Neurosci. Methods.

[46] Papousek I., Weiss E.M., Schulter G., Fink A., Reiser E.M., Lackner H.K. (2014). Prefrontal EEG alpha asymmetry changes while observing disaster happening to other people: Cardiac correlates and prediction of emotional impact. Biol. Psychol..

[47] Goedhart A.D., Van der Sluis S., Houtveen J.H., Willemsen G., De Geus E.J. (2007). Comparison of time and frequency domain measures of RSA in ambulatory recordings. Psychophysiology.

[48] Camm A.J., Malik M., Bigger J., Breithardt G., Cerutti S., Cohen R., Coumel P., Fallen E., Kennedy H., Kleiger R. (1996). Heart rate variability: Standards of measurement, physiological interpretation and clinical use. Task Force of the European Society of Cardiology and the North American Society of Pacing and Electrophysiology. Circulation.

